# KDM2B in papillomavirus-related cancer

**DOI:** 10.18632/oncoscience.434

**Published:** 2018-06-27

**Authors:** Elektra Peta, Giulia Masi, Luisa Barzon

**Affiliations:** Department of Molecular Medicine, University of Padova, 35121 Padova, Italy.

**Keywords:** human papillomavirus, miR-146a-5p, E6 and E7 oncogenes, KDM2B, stem cell

Human papillomaviruses (HPVs) are small viruses with a circular double-stranded DNA genome that specifically infect mucosal and cutaneous stratified squamous epithelia. HPV life cycle is tightly linked to the differentiation program of host epithelial cells. The virus infects proliferating cells of the basal layer of epithelia, probably targeting cells with stemness features, and exploits host replication machinery for viral DNA synthesis. As infected cells differentiate while moving up in the epithelium, viral proteins are synthesized allowing assembly of viral particles and their release with exfoliated epithelial cells.

Most HPV infections are cleared within two years; however, in some cases, infection may persist, especially with the so-called high-risk HPV types, which are drivers for malignant transformation. High-risk HPVs, especially HPV16 and HPV18, are the causative agents of virtually all cases of cervical cancer and of a proportion of other ano-genital malignancies and head and neck squamous cell carcinomas.

The hallmark of HPV-related cancers is the continuous expression of the two viral oncoproteins, E6 and E7, which are essential for maintenance of the malignant phenotype. The most relevant activities of E6 and E7 are targeting and inactivation of p53 and Retinoblastoma family proteins, respectively, thereby promoting cell proliferation, chromosomal instability, and DNA damage [1]. In addition, both E6 and E7 interact with multiple molecular targets and, correspondingly, have a broad range of biological functions. These functions include: promotion of immune evasion; E6-mediated degradation of PDZ-domain proteins, which are involved in the regulation of cell adhesion and cell polarity; E6-mediated telomerase activation; E7-mediated inactivation of p21 and cell cycle dysregulation; E7 binding to γ-tubulin and induction of centrosome abnormalities; E7 binding to various chromatin modifiers, such as histone deacetylases and methyl transferases, and modulation of host cell gene expression [1, 2].

In our recent study [3], we characterized a new molecular mechanism involved in HPV-related tumorigenesis. By microarray analysis, we identified miR-146a-5p as significantly downregulated in human keratinocytes expressing HPV16 E6 and E7.

This microRNA has immune suppressive and anti-inflammatory activities by targeting regulators in TLR signaling and cytokine production, such as *TRAF*-6 and *IRAK1*, and tumor suppressor activity by targeting genes involved in cell proliferation, migration, and metastasis formation, like *EGFR* and *NOTCH1* [4]. MiR-146a-5p is downregulated in different types of cancer, including in HPV-positive penile squamous cell carcinomas [5], where low miR-146a-5p levels have been associated with *EGFR* overexpression [6].

**Figure 1:**
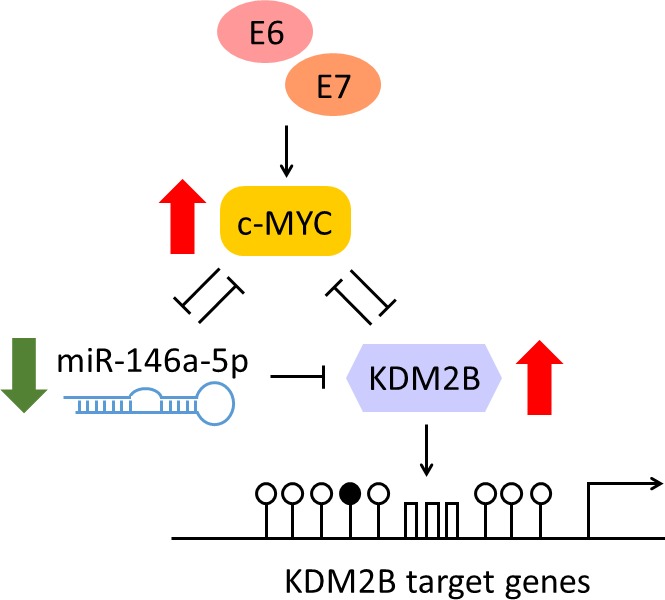
Proposed model for KDM2B upregulation by E6 and E7 of high-risk human papillomaviruses through c-MYC/miR-146-5p

In our study [3], we showed that miR-146a-5p had tumor suppressor activity in cervical cancer cells, since its forced overexpression reduced cell proliferation and migration capacity. We demonstrated that the mechanism of miR-146a-5p down-regulation in human keratinocytes and cervical cancer cells was mediated by the transcriptional repressor c-MYC, which is induced by E6 and E7 oncoproteins and has binding sites in the miR-146a promoter. Notably, we identified a new direct target for miR-146a-5p, *KDM2B/FBXL10*, which encodes a histone H3K36me2 demethylase. *KDM2B* was overexpressed in HPV16 E6/E7-positive keratinocytes, in cervical cancer cell lines, and in a subset of invasive cervical carcinomas and HPV-positive laryngeal squamous cell carcinomas, where its overexpression was associated with copy gains in the *MYC* locus. Reciprocal negative feedbacks between miR-146a-5p and c-MYC and between c-MYC and KDM2B were observed, suggesting a fine balance in the regulation of this pathway (Figure 1). Finally, we demonstrated that silencing of *KDM2B* in cervical cancer cell lines led to a decrease of cell proliferation and migration.

KDM2B is an epigenetic factor that was identified in a screening for novel oncogenes by using retroviral insertion mutagenesis in mice [7]. KDM2B is a component of the polycomb repressive complex PRC1.1 and its function depends on a zinc-finger domain, which mediates genome-wide binding to unmethylated CpG islands. Polycomb group proteins are transcription regulators that control the expression of a variety of genes involved in cell fate specification and maintenance of stem cell pools. By modulating gene expression, KDM2B induces cell proliferation, promotes the bypass of senescence and cell immortalization, and facilitates the generation of induced pluripotent stem cells by enhancing the activation of endogenous reprogramming factors [8].

In the context of HPV infection and transformation, KDM2B activity could contribute to generate a cellular milieu characterized by a proliferative state, which is exploited for viral genome replication, and by reactivation of stemness gene expression, which facilitates establishment of persistent HPV infection and the occurrence of further genetic and epigenetic events leading to malignancy.
